# Targeting Telomere Shortening in Vascular Aging and Atherosclerosis: Therapeutic Promise of *Astragalus membranaceus*

**DOI:** 10.3390/jcdd12090341

**Published:** 2025-09-04

**Authors:** Paola Canale, Maria Grazia Andreassi

**Affiliations:** 1 Health Science Interdisciplinary Center, Sant’Anna School of Advanced Studies, 56124 Pisa, Italy; 2 CNR Institute of Clinical Physiology, 56124 Pisa, Italy; mariagrazia.andreassi@cnr.it

**Keywords:** telomere dysfunction, vascular aging, *Astragalus membranaceus*, telomerase activation, atherosclerosis

## Abstract

Telomere dysfunction has emerged as a pivotal contributor to vascular senescence, a fundamental process in the pathogenesis of age-related cardiovascular diseases such as atherosclerosis. This connection underscores the therapeutic potential of targeting telomere biology to prevent or mitigate the progression of vascular aging. In this context, *Astragalus membranaceus* and its bioactive constituents, including astragaloside IV, cycloastragenol, and the commercial telomerase activator TA-65, demonstrate significant promise in attenuating vascular aging and atherosclerotic disease. These compounds exert a range of pleiotropic effects, including anti-inflammatory, antioxidant, endothelial-protective, and lipid-modulating actions, while also modulating telomerase activity and supporting telomere maintenance. This review provides an overview of the mechanistic basis underlying the anti-atherosclerotic effects of *Astragalus*-derived compounds and underscores critical key knowledge gaps. It also outlines future research directions necessary to validate their efficacy and therapeutic potential in the prevention and treatment of atherosclerosis and other age-related vascular disorders.

## 1. Introduction

Vascular senescence is now recognized as a key driver in the development and progression of atherosclerosis. Telomeres, the protective caps at the ends of chromosomes, play a crucial role in cellular aging and vascular health.

Indeed, progressive telomere attrition accelerates vascular senescence and atherosclerosis over time [1,2,3]. In endothelial and vascular smooth muscle cells, telomere shortening acts as a biological clock, triggering cells to enter replicative senescence or undergo apoptosis. Shortened telomeres initiate DNA damage responses, increase oxidative stress, and activate inflammatory signaling, fostering a pro-inflammatory secretory phenotype (SASP). This contributes to chronic low-grade inflammation within the vessel wall, exacerbating vascular dysfunction and plaque development [4,5].

Over the past few years, recent meta-analyses and Mendelian randomization studies across diverse ethnic populations have reported a significant association between shortened leukocyte telomeres and an increased risk of coronary atherosclerosis, myocardial infarction, and ischemic heart disease [6,7,8,9,10,11]. These findings provide strong evidence linking telomere length reduction to atherosclerosis progression. Since telomere shortening is closely linked to vascular aging, strategies to preserve telomere length or counteract telomere-associated dysfunction may hold significant therapeutic potential [12,13]. These approaches offer promising avenues for combating the atherosclerotic disease and promoting healthy vascular aging. Various interventions aimed at enhancing telomere length, through genetic manipulation or pharmacological means, have already demonstrated potential in delaying cellular and tissue aging [12,13].

*Astragalus*, a plant widely used in traditional Chinese medicine, has attracted considerable attention for its ability to activate telomerase and extend telomere length, making it a promising natural supplement for promoting healthy aging [14]. Recently, research on the therapeutic effects and mechanisms of *Astragalus membranaceus* (AM) and its active compounds has also gained significant interest in the cardiovascular field [15,16,17,18].

The cardioprotective effects of *Astragalus* are mediated through multiple pharmacological actions, including anti-inflammatory, antifibrotic, antioxidative, antidiabetic, immunoregulatory, and cardioprotective mechanisms, acting via numerous signaling pathways [15,16,17,18]. These properties highlight its potential as a complementary strategy for cardiovascular disease prevention and treatment.

However, its comprehensive effects on vascular aging and atherosclerosis and plausible mechanisms remain unclear.

To shed light on the mechanisms of action and promote future research, this paper reviews and discusses current evidence on the anti-atherosclerotic effects of AM and its active compounds, with a particular focus on their impact on telomere biology and vascular aging.

## 2. Overview of Telomere Biology

Telomeres are nucleoprotein structures located at the ends of chromosomes. They are essential for protecting chromosomes from degradation, fusion, and inappropriate recombination. They consist of a variable number of double-stranded telomeric repeats, 5′-(TTAAGGG)n-3′, together with a terminal region containing a single-stranded G-rich 3′ overhang. This overhang folds into higher-order structures to enhance chromosome end protection [19,20].

Telomere repeats are associated with the Shelterin multi-protein complex. This complex includes factors that bind specifically to different telomeric regions; the telomere protection 1 (Pot1)-TTP1 heterodimer binds directly to the G-strand overhang, while the double-stranded telomeric region is recognized by the telomere repeat binding factors 1 (TRF1) and the telomere repeat binding factors 2 (TRF2). In addition, these factors interact with proteins such as repressor activator protein 1 (RAP1) and TRF1-interacting nuclear protein 2 (TIN2) [20].

During DNA replication, telomeres gradually shorten in most somatic cells due to the end-replication problem. Because of this progressive shortening, telomeres function as a biological clock, with their length correlating closely with cellular aging. When telomeres become critically short, the cell activates a DNA damage response, which can lead to cellular senescence (permanent cell cycle arrest) or apoptosis (programed cell death). However, certain cells, such as stem cells, germ cells, and many cancer cells, express telomerase, a reverse transcriptase enzyme that adds telomeric repeats to chromosome ends, thereby counteracting telomere shortening and enabling continued cell division [19,20].

This enzyme comprises a catalytic subunit, telomerase reverse transcriptase (TERT), which utilizes the telomerase RNA component (TERC). TERC contains a sequence complementary to the telomeric repeats, enabling the synthesis of new telomeric DNA at the overhang [19,20].

**Figure 1:**
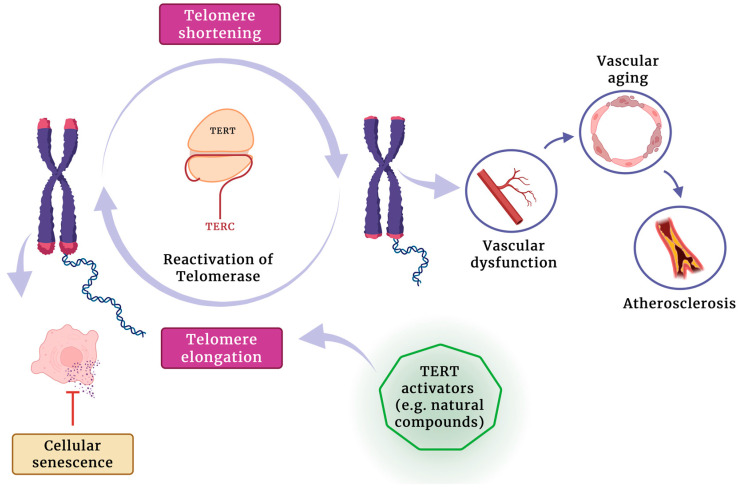
The potential role of telomerase activators in promoting telomere elongation, reducing cellular senescence, and mitigating vascular aging and atherosclerosis development.

Improving our understanding of telomere length and maintenance mechanisms, including the controlled and targeted reactivation of telomerase, offers a promising strategy for extending healthy lifespan and treating degenerative diseases by counteracting telomere-driven cellular aging (Figure 1).

## 3. *Astragalus membranaceus* and Its Active Compounds

*Astragalus* radix, the dried root of *Astragalus membranaceus*, has been used for centuries as an herbal remedy to counteract oxidative stress, inflammation, and aging [21]. The pharmacological effects of *Astragalus* radix result from a multitude of its chemical constituents. Over 200 compounds have been identified in *Astragalus*-based herbal formulations [22], the most notable being saponins, polysaccharides, and flavonoids; but it also includes components such as anthraquinones, alkaloids, amino acids, β-sitosterol, and metallic elements [21]. The main active constituents in *Astragalus* supplements are the triterpenoid saponins, mainly astragalosides. Among these, astragaloside IV is the primary active component of the medicinal *Astragalus* plant. Astragaloside IV (AS IV) is a lanolin alcohol-derived tetracyclic triterpene saponin that possesses a range of pharmaceutical properties, including anti-inflammatory, anti-insulin resistance, antitumor, and neuroprotective effects [23]. Its molecular formula is C_4_H_6_O_14_, and its structure is similar to that of steroidal drugs, with very low solubility.

Cycloastragenol (CAG) is another bioactive molecule derived from various species of the *Astragalus* genus [24]. It is an aglycone derivative of AS IV and a triterpenoid saponin compound formed through the hydrolysis of AS IV [24].

TA-65 is a patented, natural, encapsulated form of cycloastragenol that has gained attention as a safe and effective dietary supplement for promoting healthy aging, with no reported toxicity [25]. Another active ingredient of *Astragalus* radix is *astragalus* polysaccharide (APS), a heteropolysaccharide with a complex chemical structure and water solubility [17]. It contains heteropolysaccharides, dextran, and fractions of both neutral and acidic polysaccharides, which have demonstrated significant immunomodulatory effects and anticancer activity [26].

**Figure 2:**
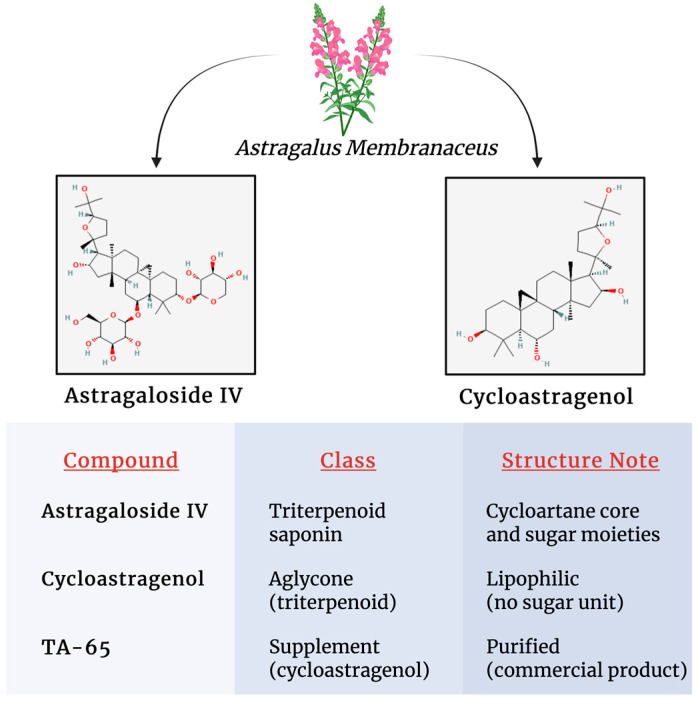
Structural characteristics and detailed physicochemical properties of major *Astragalus* constituents.

The structure and the detailed physicochemical properties of the main *Astragalus*-derived compounds are summarized in Figure 2.

## 4. Anti-Senescence Mechanisms and Anti-Aging-Related Effects of *Astragalus* and Its Chemical Constituents

### 4.1. Preclinical and Animal Evidence

Several studies have demonstrated that CAG and/or AS IV can delay or mitigate cellular senescence and apoptosis under various experimental conditions [27,28]. Both compounds have been identified as telomerase-stimulating agents [27,28]. Telomerase activity is crucial for maintaining the proliferative capacity and longevity of somatic cells [29], but it declines significantly in adult organisms compared to neonates [29].

The CAG has been shown to enhance telomerase activity in neuronal cells via activation of the cyclic adenosine monophosphate response element-binding protein (CREB), a key transcription factor required for upregulating TERT expression and downstream telomerase function [28]. Similarly, AS IV has been reported to increase telomerase expression by modulating several intracellular pathways, including the Mitogen-Activated Protein Kinase (MAPK), Janus Kinase/Signal Transducer and Activator of Transcription (JAK/STAT), and CREB signaling cascades [24]. In addition, AS IV is capable of activating telomerase in a range of cell types, particularly in mouse embryonic fibroblasts (MEFs, G3 Terc^+/−^) and hematopoietic progenitor cells. Systemic supplementation of AS IV has been shown to boost TERT activation in various tissues, including the brain, liver, heart, lungs, and bone marrow, thereby alleviating telomere attrition in aged mice [30,31].

Furthermore, co-administration of AS IV and CAG has been shown to enhance telomerase activity via activation of the proto-oncogene tyrosine-protein kinase Src/Mitogen-Activated Protein Kinase Kinase/Extracellular Signal-Regulated Kinase (Src/MEK/ERK) signaling cascade [27]. A study demonstrated that both CAG and AS IV play a significant role in preventing high glucose (HG)-induced intervertebral disc degeneration by attenuating cellular senescence, both replicative and stress-induced premature senescence. These compounds may also inhibit apoptosis in nucleus pulposus cells by upregulating telomerase activity and promoting telomere elongation [23]. The activation mediated by CAG is primarily associated with the MAPK and Protein Kinase B pathways (Akt). Specifically, Akt enhances telomerase enzymatic activity by promoting post-translational phosphorylation of the TERT subunit [32].

The CAG has been found to induce regulated protein kinase (ERK) phosphorylation in Human Embryonic Kidney 293 cells (HEK293) and neonatal keratinocytes by engaging the Src/MEK/ERK pathway [27].

Like CAG and AS-IV, TA-65 is a small-molecule telomerase activator, commercially available on the market since 2008. Since its introduction, it has been investigated in preclinical and early clinical work for its potential role in promoting telomere maintenance and cellular health [33]. For instance, it has been shown to increase average telomere length and reduce both the frequency of critically short telomeres and DNA damage in haplo-insufficient mouse embryonic fibroblasts (MEFs, G3 Terc^+/−^). Indeed, these cells exhibit telomere shortening due to their possession of a single copy of the telomerase RNA component gene [30].

In addition, the dietary supplementation of 25 mg/kg per day of TA-65 is capable of inducing an improvement of certain health-span indicators, including glucose tolerance, osteoporosis and skin fitness, without significantly increasing global cancer incidence [30].

Moreover, TA-65 showed protective effects in mouse models of chronic obstructive pulmonary disease (COPD) and cigarette-smoke damage. This protective effect was associated with a decreased expression of the pro-fibrotic cytokine transforming growth factor beta 1 (TGF-β1) in the small airway walls of CS-exposed mice, together with a protection against fibroblast-to-myofibroblast differentiation in response to TGF-β1 in lung primary fibroblast in vitro [34].

### 4.2. Human Investigation Studies

Human studies have also supported these in vitro findings, indicating that TA-65 can positively influence telomere length in healthy individuals [33].

A study of 117 relatively healthy individuals aged 53–87 years and positive for cytomegalovirus found that a low dose of TA-65 (250 U) significantly increased telomere length over 12 months, whereas participants in the placebo group experienced significant telomere shortening [35].

A placebo-controlled study recruiting a large number of healthy adults (500) showed that oral intake of TA-65 across all doses (100 U, 250 U, and 500 U) for nine months significantly decreased CD8^+^CD28^−^ T cells, suggesting a potential benefit by decreasing senescent T cells [36].

Similarly, in a randomized, double-blind, placebo-controlled six-month trial involving 40 healthy volunteers, participants taking an *Astragalus*-based supplement exhibited significantly increased median and short telomere lengths, while no change was observed in the placebo group [37].

Although these data suggest that telomerase therapies and pharmacological interventions can counteract the effects of telomere attrition, their role in vascular biology and atherosclerosis is still largely unknown.

## 5. *Astragalus* in Vascular Cells and Atherosclerosis

### 5.1. Effects on HUVEC

Endothelial cells, which are essential elements of the arterial intima, play a key role in regulating vascular function and preserving internal homeostasis.

Endothelial dysfunction, characterized by reduced nitric oxide production, oxidative stress, and proinflammatory signaling, drives atherosclerosis by promoting plaque formation, immune cell recruitment, and lipid accumulation in the arterial wall [38].

As previously mentioned, telomere shortening is strongly associated with vascular dysfunction by promoting cellular senescence, which impairs vascular repair, elevates oxidative stress, and contributes to the development of atherosclerosis [1,2,3]

Numerous studies have demonstrated the protective effects of AS IV against vascular endothelial dysfunction [39], although evidence regarding its influence on telomere biology in endothelial cells remains lacking. In vitro studies have shown that AS IV at 80 μmol/L promotes proliferation and angiogenic activity in human umbilical vein endothelial cells (HUVECs), primarily through the suppression of phosphatase and tensin homolog (PTEN) expression and activation of the Phosphoinositide 3-Kinase (PI3K)/Akt signaling pathway [40]. In addition, Zhang et al. showed that a low concentration of AS IV (0.25 μM) facilitates capillary-like tube formation in HUVECs through PI3K/Akt pathway activation and upregulation of hypoxia-inducible factor 1-alpha (HIF-1α), suggesting a role in neovascularization [41].

In agreement with these data, Wang et al. demonstrated that AS IV (10, 40, and 120 μM) significantly promotes proliferation, migration, and tube formation in HUVECs via ERK1/2 phosphorylation and JAK2/STAT3 pathway activation [42].

AS IV also exhibits antioxidant properties in oxidative injury models. Xu et al. reported that AS IV (20–100 μmol/L) increased nitric oxide (NO) bioavailability in H_2_O_2_-stimulated HUVECs by inhibiting reactive oxygen species (ROS)/Nuclear Factor kappa-light-chain-enhancer of activated B (NF-κB) signaling and reducing endothelial nitric oxide synthase (eNOS) uncoupling [43]. Zhu et al. further reported that AS IV at concentrations of 10, 20, and 50 μM significantly enhances oxidized Low Density Lipoprotein (LDL)-induced HUVECs migration and motility while suppressing the generation of ROS and NADPH oxidase (NOX). These effects are mediated by activation of the nuclear factor erythroid 2-related factor 2 (NRF2)/heme oxygenase-1 (HO-1) signaling axis, a key regulator of oxidative stress responses [44].

Qiu et al. observed that AS IV at doses of 50 and 100 mg/mL mitigated Homocysteine (HCY)-induced endothelial dysfunction, a known risk factor for atherosclerosis, by reducing ROS accumulation and enhancing superoxide dismutase (SOD) activity, thereby restoring cellular homeostasis [45]. Similar observations were reported by Shao et al. They investigated the role of AS IV in a circular RNA-mediated regulatory mechanism using an in vitro atherosclerosis model in which HUVECs were exposed to ox-LDL. AS IV (100 μM) reduced apoptosis, oxidative stress, and inflammation, while enhancing cell viability and migration. These effects were mediated through modulation of the circ_0000231/miR-135a-5p/Chloride Intracellular Channel 4 (CLIC4) axis [46].

Complementary to these findings, Chen et al. explored the molecular basis of AS-IV’s protective effects against ox-LDL-induced endothelial injury. AS IV treatment inactivated the NF-κB pathway by regulating the histone deacetylase 9 (HDAC9), leading to reduced apoptosis, oxidative stress, and inflammatory signaling [47].

Together, these findings highlight AS IV as a multifunctional compound with significant potential in the prevention and treatment of endothelial dysfunction and atherosclerosis through different molecular pathways.

### 5.2. Effects on VSMCs

Like endothelial cells, vascular smooth muscle cells (VSMCs) play a key role in the development and progression of atherosclerosis by contributing to plaque formation, vascular wall remodeling, and the regulation of inflammation and calcification [48].

However, studies investigating the protective effects of *Astragalus membranaceus* in this context remain limited. Zhang et al. reported that AS IV at a concentration of 10 μM inhibited Angiotensin II-induced proliferation of A10 cells (a rat VSMC line) by reducing the cyclin-dependent kinase 2 (CDK2) activity [49]. CDK2 is essential for the transition from the G1 to the S phase of the cell cycle, as well as for regulating the G2 phase, thereby promoting cell proliferation [50].

Further supporting evidence comes from Lu et al., who found that AS IV (50 μg/mL) restored Adenosine Triphosphate (ATP) production in Angiotensin II-induced VSMCs. Additionally, AS IV reversed mitochondrial dysfunction by enhancing oxygen consumption rates, increasing mitochondrial membrane potential, and boosting mitochondrial DNA content. These mitochondrial improvements were accompanied by a reduction in ROS production, increased SOD activity, and stimulation of mitochondrial biogenesis and mitophagy [51].

Consistent with these findings, Li et al. reported that AS IV attenuated senescence in bleomycin-induced VSMCs by restoring mitochondrial membrane potential, improving mitochondrial integrity, and promoting mitophagy. This anti-senescent effect was mediated via Parkin upregulation, highlighting the relevance of AS-IV-induced mitophagy in preserving VSMCs homeostasis [52].

However, in another study, Song et al. demonstrated that AS IV (50 μg/mL) can inhibit the autophagy and mineralization of VSMCs by increasing the expression of long non-coding RNA H19 (lncRNA H19) and decreasing the expression of dual-specificity phosphatase 5 (DUSP5) [53].

**Figure 3:**
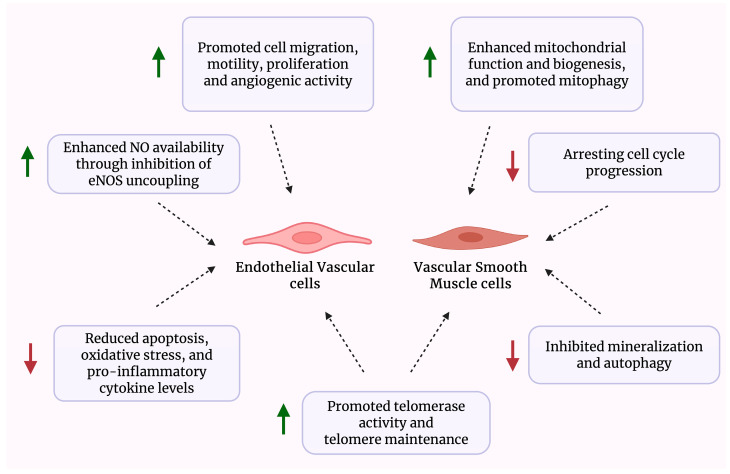
Schematic overview of the biological effects of *Astragalus*-derived compounds on vascular cells.

Table 1 presents a summary of studies investigating the beneficial effects of astragaloside IV, whereas Figure 3 illustrates a schematic overview of the effects of *Astragalus*-derived compounds on vascular cells.

### 5.3. In Vivo Effects on Atherosclerosis and Plaque

Several in vivo studies have investigated the anti-atherosclerotic effects of astragaloside IV, particularly in murine models of atherosclerosis (Table 2). These studies have provided valuable insights into the compound’s pharmacological mechanisms and its potential for modulating plaque development and vascular inflammation. In an in vivo model of atherosclerosis, ApoE^−/−^ mice were fed a high-fat diet (HFD) ad libitum and treated daily with AS IV (25 mg/kg) for eight weeks. This model exhibited increased autophagy and mineralization in VSMCs of the thoracic aorta. AS IV administration significantly attenuated these effects, suggesting a protective role against VSMCs dysfunction [53].

Similarly, Wang et al. reported that AS IV treatment at 40 mg/kg reduced lipid-rich areas in atherosclerotic plaques of ApoE^−/−^ mice. This was accompanied by increased collagen content and fibrous cap thickness, effects linked to regulation of the PI3K/Akt and Toll-Like Receptor 4 (TLR4)/NF-κB pathways, inhibition of Matrix Metalloproteinase-9 (MMP-9) expression, and anti-inflammatory activity [54]. In LDLR^−/−^ mice, Zhang et al. demonstrated that AS IV mitigated atherosclerosis by targeting the MAPK/NF-κB signaling pathway. This intervention reduced NF-κB p65 expression in the aortic root and decreased inflammatory cytokine levels in serum, aortic, and liver tissues. The anti-inflammatory effects involved inhibition of MAPK components (JNK, ERK1/2, and p38), suppression of NF-κB signaling, and reduced phosphorylation of inflammatory proteins, including inducible Nitric Oxide Synthase (iNOS), Vascular Cell Adhesion Molecule-1 (VCAM-1), and Interleukin-6 (IL-6) [55].

Moreover, Sun et al. found that daily AS IV administration at 20 mg/kg in rats upregulated Peroxisome Proliferator-Activated Receptor gamma (PPAR-γ) through NF-κB inhibition, resulting in decreased serum concentrations of ox-LDL, Tumor Necrosis Factor alpha (TNF-α), IL-6, and IL-18, thereby suppressing atherosclerosis progression [56]. Similarly, Qin et al. showed that AS IV at 40 mg/kg/day reduced atherosclerotic severity in ApoE^−/−^ mice via modulation of the stromal-cell-derived factor-1 (SDF-1)/CXC chemokine receptor 4 (CXCR4) pathway [57]. Interestingly, a meta-analysis of 22 animal I/R model studies showed consistent reduction in infarct size, affirming strong reproducibility across models [58].

Beyond direct vascular and anti-inflammatory effects, modulation of lipid metabolism represents another key mechanism by which *Astragalus* compounds may protect against atherosclerosis. Hypercholesterolemia is a major driver of atherosclerosis. In experimental models of diet-induced hypercholesterolemia, APS significantly lowered plasma cholesterol, triglycerides, and LDL-cholesterol, while also reducing hepatic lipid accumulation, enhancing fecal bile acid and sterol excretion, inhibiting intestinal cholesterol absorption, and upregulating hepatic cholesterol-7α-hydroxylase and LDL receptor expression [59]. In parallel, inflammatory pathways contribute to lipid dysregulation; TNF-α impairs reverse cholesterol transport by downregulating ATP-binding cassette transporter A1 (ABCA1), promoting foam cell formation [60]. APS counteracts this process by restoring ABCA1 expression, promoting cholesterol efflux, and suppressing NF-κB activation, thereby limiting inflammation-driven lipid accumulation [61]. These findings suggest that, *Astragalus*-derived compounds such as AS IV and APS may exert complementary anti-atherosclerotic actions through lipid-lowering and cholesterol-transport modulation.

### 5.4. Human Clinical Studies

Limited but promising clinical data have also demonstrated improvements in cardiac function and oxidative stress markers in acute myocardial infraction and ischemic heart disease.

Indeed, some preliminary Chinese observational studies have reported positive effects of *Astragalus* on symptoms such as dyspnea, chest pain, and angina, alongside improvements in electrocardiographic parameters and cardiac output in patients with ischemic heart disease [62,63].

A double-blind, randomized, placebo-controlled trial in which patients with metabolic syndrome were allocated to consume either 16 mg daily of a TA-65 supplement or a placebo for 12 weeks showed that there was an improvement in risk factors for cardiovascular disease, with reduced inflammatory levels (low TNF-α levels) and a parallel reduction in body mass index, waist circumference, and atherosclerotic ratio LDL-C/High-Density Lipoprotein (HDL) [64].

More recently, the Telomerase ACTivator to reverse Immunosenescence in Acute Coronary Syndrome (TACTIC) study, a double-blinded, randomized controlled trial, evaluated for the first time whether TA-65 can reduce immune cell aging in patients following myocardial infarction. The study demonstrated that one year of treatment with TA-65 significantly enhanced telomerase activity in immune cells, resulting in a marked reduction in systemic inflammatory markers such as IL-6 and TNF-α. Additionally, it improved lymphocyte proliferation and decreased signs of immune senescence. These findings highlight the promising therapeutic role for TA-65 in modulating post-infarction inflammation and immune function [65].

## 6. Conclusions and Perspectives

Telomere dysfunction has emerged as a critical contributor to vascular senescence, a key process in the development of age-related cardiovascular diseases such as atherosclerosis. This strong association highlights the potential of therapeutic strategies that enhance telomere biology to prevent or slow the progression of atherosclerosis.

Within this context, *Astragalus membranaceus* and its bioactive compounds, including astragaloside IV, cycloastragenol, and the commercially available telomerase activator TA-65 demonstrate considerable promise in reducing vascular aging and atherosclerosis. Further supporting their relevance in cardiovascular disease, astragaloside IV and related compounds have also demonstrated protective roles in a range of cardiac pathologies, such as heart failure, hypertrophy, fibrosis, and ischemia–reperfusion injury.

As discussed above, these agents exert pleiotropic effects on vascular biology, including anti-inflammatory, antioxidant, endothelial-protective, and lipid-regulating actions. Mechanistically, they influence key signaling pathways such as PI3K/Akt and ROS/NF-κB, while also modulating telomerase activity and telomere maintenance, both increasingly recognized as central regulators of vascular senescence and plaque instability.

Despite these encouraging findings, the precise molecular targets of *Astragalus membranaceus*-derived compounds in vascular cells remain only partially understood. The reactivation of endogenous telomerase activity may represent a fundamental mechanism underlying their atheroprotective effects. However, the direct involvement of telomere–telomerase regulation in mediating these benefits remains speculative.

A promising avenue for future research is to determine whether shelterin-related complexes act as intermediaries or modulators of the effects of *Astragalus membranaceus* and its derivatives on telomerase function. The shelterin complex, a group of six telomere-binding proteins, TRF1, TRF2, POT1, TIN2, telomere protection protein 1 (TPP1) and RAP1, is essential for telomere protection and serves as a key negative regulator of telomerase access to chromosome ends [66]. A deeper understanding of the specific roles and regulatory mechanisms of each shelterin component could reveal how *Astragalus* compounds interact with telomere biology and identify novel molecular targets for the controlled activation of telomerase in vascular cells.

In addition, telomeric repeat-containing RNA (TERRA), a long non-coding RNA transcribed from telomeric regions, has emerged as a significant regulator of telomere integrity and telomerase activity [67]. Future studies should investigate whether *Astragalus membranaceus* influences TERRA expression or its interaction with telomerase, and how this may contribute to telomere homeostasis and vascular cell aging.

Moreover, it is becoming increasingly evident that telomerase exerts multiple non-canonical functions beyond telomere length maintenance, including roles in DNA damage response, transcriptional regulation, and mitochondrial function.

Notably, recent studies suggest that astragaloside IV also modulates mitochondrial biogenesis and mitophagy, particularly in VSMCs exposed to senescence-inducing stimuli such as angiotensin II. AS IV promotes Parkin-mediated mitophagy and supports mitochondrial homeostasis, factors now recognized as critical in the regulation of vascular aging [52]. Given the well-established bidirectional crosstalk between telomere dysfunction and mitochondrial decline, exploring how *Astragalus*-derived compounds affect the telomere–mitochondrion axis may provide novel mechanistic insights into their vasculoprotective properties.

Consequently, future studies should elucidate the impact of *Astragalus membranaceus* on telomerase-associated pathways to better understand its full therapeutic potential in the context of vascular aging.

To elucidate these relationships, future in vitro studies should investigate the effects of astragaloside IV, cycloastragenol, and TA-65 on telomerase activity, telomere length maintenance, mitochondrial dynamics, and senescence markers in both HUVECs and VSMCs, particularly under conditions of oxidative or inflammatory stress.

Although preliminary preclinical and clinical data are encouraging, comprehensive randomized controlled trials and long-term safety assessments are still required.

Future research should also aim to define dose–response relationships, explore potential synergistic effects with standard therapies, and validate robust biomarkers of vascular rejuvenation.

Additionally, given that these compounds act as telomerase activators and that telomerase reactivation is implicated in more than 80% of human tumors [68], the potential carcinogenic risk cannot be ruled out and should be rigorously assessed before considering clinical applications.

These efforts will be essential for establishing therapeutic efficacy, safety, and clinical utility of *Astragalus*-derived compounds in the prevention and treatment of atherosclerosis and other age-related vascular disorders.

## Figures and Tables

**Table 1:** Studies investigating the beneficial effects of astragaloside IV on vascular cells.

Author	Cell Type	Models	Observed Effects	Proposed Mechanism
Cheng et al. [40]	HUVECs	—	Promotes proliferation and angiogenic activity	Suppression of PTEN expression and activation of the PI3K/Akt pathway
Zhang et al. [41]	HUVECs	Hypoxia exposure	Stimulated angiogenic activity	Activation of PI3K/Akt pathway and upregulation of HIF-1α
Wang et al. [42]	HUVECs	—	Enhanced endothelial cell proliferation, migration, and tube formation	Activation of ERK1/2 and JAK2/STAT3 pathways
Xu et al. [43]	HUVECs	H_2_O_2_-induced oxidative injury	Enhanced NO availability through inhibition of eNOS uncoupling	Inhibition of ROS/NF-κB pathway
Zhu et al. [44]	HUVECs	ox-LDL exposure	Promoted cell migration and motility; decreased ROS and NOX	Activation of Nrf2/HO-1 axis
Qiu et al. [45]	HUVECs	Homocysteine-induced endothelial injury	Reduced ROS, increased SOD activity, and improved cellular redox balance	Antioxidant activity and redox homeostasis restoration
Shao et al. [46]	HUVECs	ox-LDL exposure	Decreased apoptosis, oxidative stress, and pro-inflammatory cytokine	Modulation of circ_0000231/miR-135a-5p/CLIC4 axis
Chen et al. [47]	HUVECs	ox-LDL stimulation	Reduced apoptosis and oxidative stress	Inactivation of the NF-κB pathway through the regulation of HDAC9
Zhang et al. [49]	Rat VSMCs	Angiotensin II stimulation	Arresting cell cycle progression	Downregulation of CDK2 activity
Lu et al. [51]	Rat VSMCs	Angiotensin II stimulation	Enhanced mitochondrial function and biogenesis	Increased SOD activity and reduction in ROS
Li et al. [52]	VSMCs	Bleomycin-induced senescence	Restored mitochondrial integrity, promoted mitophagy	Parkin-mediated mitophagy
Song et al. [53]	VSMCs	Calcification model	Inhibited mineralization and autophagy	Upregulation of lncRNA H19 and inhibition of DUSP5 expression

**Table 2:** Studies investigating the beneficial effects of astragaloside IV in animal models of atherosclerosis.

Author	Animal Model	Observed Effects	Proposed Mechanism
Song et al. [53]	ApoE^−/−^ mice + HFD	Reduced autophagy and mineralization in the thoracic aorta	Protective effect against VSMCs dysfunction
Wang et al. [54]	ApoE^−/−^ mice + HFD	Decreased lipid-rich plaque areas; increased collagen content and fibrous cap thickness	Regulation of PI3K/Akt and TLR4/NF-κB pathways; inhibition of MMP-9; anti-inflammatory activity
Zhang et al. [55]	LDLR^−/−^ mice + HFD	Reduced NF-κB p65 expression and serum/aortic/liver cytokine levels	Inhibition of MAPK/NF-κB pathway and reduced iNOS, VCAM-1, and IL-6 phosphorylation
Sun et al. [56]	Rats + HFD	Decreased serum ox-LDL, TNF-α, IL-6, and IL-18; suppressed plaque progression	NF-κB inhibition; upregulation of PPAR-γ
Qin et al. [57]	ApoE^−/−^ mice + HFD	Reduced severity of atherosclerosis	Modulation of the SDF-1/CXCR4 pathway
Zheng et al. [58]	Multiple preclinical models	Anti-inflammatory and antioxidant actions in myocardial I/R injury	Multiple mechanisms (regulation of inflammation and oxidative stress)

## Abbreviations

The following abbreviations are used in this manuscript:

ABCA1	ATP-Binding Cassette Transporter A1
Akt	Protein Kinase B pathway
AM	*Astragalus membranaceus*
APS	*Astragalus* Polysaccharide
AS IV	Astragaloside IV
ATP	Adenosine Triphosphate
CAG	Cycloastragenol
CDK2	Cyclin-Dependent Kinase 2
CLIC4	Chloride Intracellular Channel 4
COPD	Chronic Obstructive Pulmonary Disease
CREB	cAMP Response Element-Binding Protein
CXCR4	CXC Chemokine Receptor 4
DUSP5	Dual Specificity Phosphatase 5
eNOS	Endothelial Nitric Oxide Synthase
ERK	Regulated Protein Kinase
HCY	Homocysteine
HDAC9	Histone Deacetylase 9
HDL	High-Density Lipoprotein
HEK293	Human Embryonic Kidney 293 Cells
HFD	High-Fat Diet
HIF-1α	Hypoxia-Inducible Factor 1-alpha
HO-1	Heme Oxygenase-1
HUVECs	Human Umbilical Vein Endothelial Cells
IL-6 /IL-18/IL-1β	Interleukin-6/-18/-1 beta
iNOS	inducible Nitric Oxide Synthase
JAK/STAT	Janus Kinase/Signal Transducer and Activator of Transcription
LDL	Low Density Lipoprotein
lncRNA H19	Long non-coding RNA H19
MAPK	Mitogen-Activated Protein Kinase
MEFs	Mouse Embryonic Fibroblasts
MEK	Mitogen-Activated Protein Kinase Kinase
MMP-9	Matrix Metalloproteinase-9
NF-κB	Nuclear Factor kappa-light-chain-enhancer of activated B cells
NO	Nitric Oxide
NOX	NADPH Oxidase
NRF2	Nuclear factor erythroid 2-Related Factor 2
PI3K	Phosphoinositide 3-Kinase
POT1	Telomere Protection 1
PPAR-γ	Peroxisome Proliferator-Activated Receptor gamma
PTEN	Phosphatase and Tensin Homolog
RAP1	Repressor Activator Protein 1
ROS	Reactive Oxygen Species
SASP	Senescence-Associated Secretory Phenotype
SDF-1	Stromal cell-Derived Factor-1
SOD	Superoxide Dismutase
Src	Proto-oncogene tyrosine-protein kinase Src
TA-65	Telomerase Activator-65
TERC	Telomerase RNA Component
TERRA	Telomeric Repeat-Containing RNA
TERT	Telomerase Reverse Transcriptase
TGF-β1	Transforming Growth Factor Beta 1
TIN2	TRF1-Interacting Nuclear Protein 2
TLR4	Toll-Like Receptor 4
TNF-α	Tumor Necrosis Factor-alpha
TPP1	Telomere Protection Protein 1
TRF1	Telomere Repeat Binding Factors 1
TRF2	Telomere Repeat Binding Factors 1
VCAM-1	Vascular Cell Adhesion Molecule 1
VSMCs	Vascular Smooth Muscle Cells
